# Facilitation of allocentric coding by virtue of object-semantics

**DOI:** 10.1038/s41598-019-42735-4

**Published:** 2019-04-18

**Authors:** Harun Karimpur, Yaniv Morgenstern, Katja Fiehler

**Affiliations:** Experimental Psychology, Justus Liebig University, Giessen, Germany

**Keywords:** Cognitive neuroscience, Perception, Spatial memory, Human behaviour

## Abstract

In the field of spatial coding it is well established that we mentally represent objects for action not only relative to ourselves, egocentrically, but also relative to other objects (landmarks), allocentrically. Several factors facilitate allocentric coding, for example, when objects are task-relevant or constitute stable and reliable spatial configurations. What is unknown, however, is how object-semantics facilitate the formation of these spatial configurations and thus allocentric coding. Here we demonstrate that (i) we can quantify the semantic similarity of objects and that (ii) semantically similar objects can serve as a cluster of landmarks that are allocentrically coded. Participants arranged a set of objects based on their semantic similarity. These arrangements were then entered into a similarity analysis. Based on the results, we created two semantic classes of objects, natural and man-made, that we used in a virtual reality experiment. Participants were asked to perform memory-guided reaching movements toward the initial position of a target object in a scene while either semantically congruent or incongruent landmarks were shifted. We found that the reaching endpoints systematically deviated in the direction of landmark shift. Importantly, this effect was stronger for shifts of semantically congruent landmarks. Our findings suggest that object-semantics facilitate allocentric coding by creating stable spatial configurations.

## Introduction

Grabbing a cup of coffee, reaching for the cookie jar, or switching on the light: in our everyday lives, we seamlessly interact with objects in our environment. What seems so easy is the result of various computations we have to make in a fraction of a second. For example, we need to perceive the object, locate it relative to ourselves and the environment and compute the motor plan before we execute the reaching movement. This study focuses on how we spatially represent *objects for action*.

Spatial representations help us to locate objects in space, even in situations when only sparse or no visual information is available. Take the example of a bedside lamp. When we wake up in the middle of the night and want to turn on the lamp, we usually know where to reach. Given that we toss and turn in bed and therefore our own position is not stable, it is likely that we have built some spatial representation of the lamp *relative* to other objects or landmarks in the environment, e.g., the corner of the bed. When we want to systematically refer to these relations, we speak of an allocentric Frame of Reference (FoR)^1,2^. This can be distinguished from an egocentric FoR, where objects are represented relative to the observer, e.g., body or gaze.

Humans represent objects for action in an egocentric, predominantly gaze-centered FoR^3,4^. However, allocentric FoR also contribute to spatial coding for action^5^, leading to improved movement precision and accuracy in online and delayed reaching^6–8^. The presence of landmarks reduces but does not fully cancel gaze-dependent reaching errors suggesting that humans integrate egocentric and allocentric FoR^9,10^, probably in a statistically optimal manner^11^.

While previous studies mainly applied simplistic, abstract stimuli, recent work addressed this shortcoming by making use of naturalistic complex scenes^12–18^. For example, in the study of Fiehler *et al*.^12^, participants freely encoded photographic images of a breakfast scene in which six breakfast items were placed on a table and served as potential reach targets. After a short delay, the scene briefly reappeared (test scene) with one object (target object) missing. The crucial manipulation here was that in some conditions, objects in the test scene were horizontally shifted. On a blank response screen participants were then asked to reach to the remembered target position. If participants encoded the target object in a purely egocentric FoR, a shift of objects in the scene should not systematically affect the reaching endpoints. If participants encoded the target object in an allocentric FoR, i.e. relative to the other objects, the reaching endpoints should deviate in the direction of object shift. The authors found evidence for the latter hypothesis suggesting that humans represent objects for action in an allocentric FoR. The effect of the object shifts on reaching endpoints was about 45% leaving room for the contribution of other allocentric and egocentric FoR. This result was further supported for reaching movements in depth in both reality^18^ and virtual reality settings^14^.

Given the plethora of environmental information surrounding us, the question arises as to which allocentric cues we select for coding objects for action. Several factors such as task relevance and scene coherence have been identified facilitating the use of allocentric information. For example, the influence of object shift on reaching endpoints was stronger when the shifted objects served as potential reach targets and therefore were task-relevant^13^. This influence increased with the number of shifted task-relevant objects. Shifts of task-irrelevant objects hardly affected reaching behavior. Task relevance has also been shown to modulate memory performance in natural search tasks in addition to actual object interaction^19–21^. Moreover, breaking down the spatial coherence of a scene by shifting objects in opposite directions strongly reduced the effect of object shift^15^. This could be explained by reduced spatial reliability of allocentric information resulting in a stronger reliance on egocentric or other more stable allocentric FoR. In summary, previous work on memory-guided reaching examined the influence of high-level factors and low-level grouping. Missing, however, is the influence of object-semantics on spatial coding for action for several reasons.

First, it is suggested that we combine egocentric and allocentric information in a statistically optimal manner whilst taking into account the reliability of landmarks or landmark configurations^11,17^. Hypothetically, such reliable configurations or groupings could be based on various factors such as Gestalt principles or semantics. Imagine a table on which we place three fruits and three tools. The grouping hypothesis, initially formulated by Yantis^22^, states that humans mentally represent scene configurations as a virtual polygon (e.g., a triangle of fruits) with the vertices being the initial locations of objects (e.g., each corner of the triangle represents the location of a fruit). Objects that are semantically similar to some and dissimilar to others could facilitate grouping and therefore allocentric coding. This is further supported by findings suggesting that one of the most natural ways of representing an object context is in terms of how it is related to other objects^23,24^. Thus, semantics seem to contribute in establishing object context that can further facilitate the formation of spatial configurations. Such semantic facilitations could already be demonstrated in object identification when familiar functionally-related objects were present^25^. Second, the kinematics in reaching and grasping tasks are affected by object-semantics^26–28^. For example, printing the word “LARGE” or “SMALL” on objects led to larger or smaller grip aperture early in the reach^26^. We conclude that object-semantics could be a mean of efficient coding and incorporated into the motor plan.

The evidence presented hitherto suggests that object-semantics could facilitate spatial coding. Here we investigate how object-semantics influence the extent to which we integrate allocentric information for reaching. This requires a controlled manipulation of object-semantics. Intuitively, one would agree that “An apple is more similar to a pear than it is to a tennis ball”. But how can we quantify such *semantic similarity*? While these exemplary objects share certain characteristics (e.g., shape) they are obviously different with respect to other characteristics (e.g., functionality). Despite these differences we can group the objects in terms of such characteristics that span a continuum, where some sets of characteristics instantiate the semantic object category they belong to. This idea goes back to Tversky and Hemenway (1983)^29^ who proposed that shared characteristics (“part configuration”) are “diagnostic of the preferred level in object categories […] Thus, in the case of objects, natural and manufactured, part configuration seems to underlie the coincidence of the basic level determined perceptually and the basic level determined behaviorally.” (p. 133). Since many factors can contaminate semantic grouping of objects, we chose a computational approach using similarity analysis to find preferred groupings for a new set of objects. In the following we will report how we (i) quantified semantic similarity and (ii) how we used these results to show that humans take semantic information into account when encoding spatial relations of objects for action.

## Results

### Quantifying semantic distances

**Figure 1:**
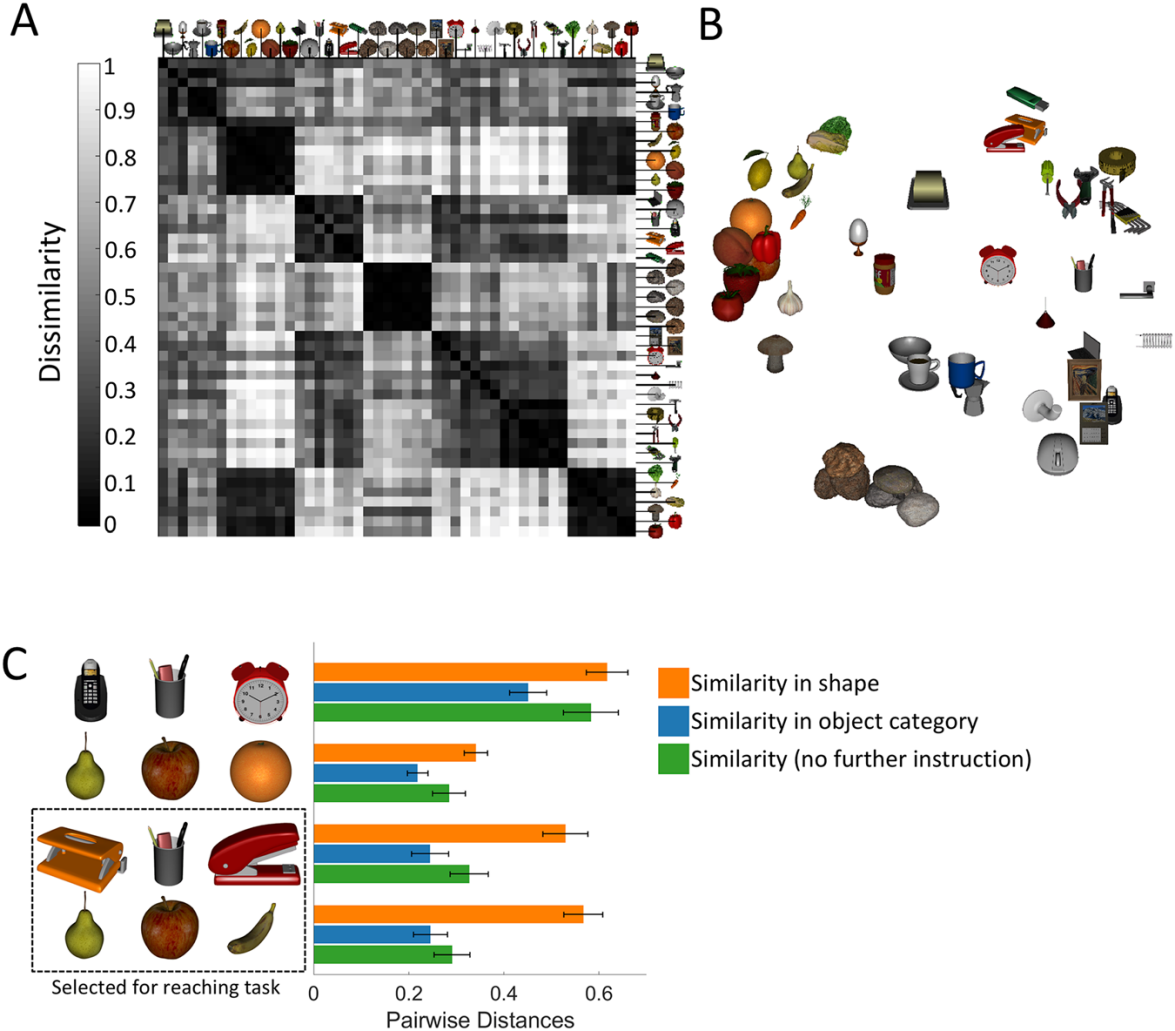
Object arrangements and representation dissimilarity matrix (RDM) for the pooled data. For each pair of objects, the RDM (**A**) codes dissimilarity. The objects have been arranged in (**B**) such that their pairwise distances approximately reflect the distances in the RDM (multidimensional scaling; dissimilarity: distances, criterion: metric stress). The data in (**A**,**B**) are for similarity arrangements where participants were not instructed to use any particular similarity criterion. In separate arrangements, participants were instructed to arrange the objects based on object category and on shape (see Supplementary Figs S1 and S2). (**C**) We selected natural and man-made objects for the reaching task whose pairwise distances in the arrangement task were near in the object category and no instruction arrangements, but farther away in shape similarity. Error bars show standard error from the mean. Stove top espresso maker printed with permission by Jono Moles under a CC BY open access license.

We used the multi-arrangement method by Kriegeskorte and Mur (2012)^30^ that was developed for Representational Similarity Analyses (RSA^31^) to quantify the semantic distances for our selected set of objects. To do this, participants performed similarity arrangements over the 49 objects without using a specified similarity criterion. We assume that objects are represented as point in a metric, high-dimensional psychological feature space, where distances between stimuli as estimated by similarity arrangements will be related to their semantic similarity; nearer distances correspond to objects that are in the same class. Figure 1A shows the dissimilarities (in terms of distances) assembled as a Representational Dissimilarity Matrix (RDM), with a height and width corresponding to the number of objects, and symmetric along the diagonal. Consistent with our assumption, the RDM pooled across participants (*n* = 15) shows that objects from similar semantic categories (e.g., rocks, fruits, vegetables, office supplies) have smaller dissimilarities (Fig. 1A; dark regions). This result can be visualized by arranging the objects using multidimensional scaling (MDS) such that the pairwise distances approximately reflect the distances in the RDM (Fig. 1B).

We also had participants make arrangements based on similarity in shape and object category (see Supplementary Figs S1 and S2, respectively). For any group of objects (e.g., fruits) that we were about to choose for the reaching task (see next section), these additional arrangements helped us to ensure that the selected objects were heterogeneous in shape but homogenous with respect to the object category they belonged to. Otherwise any grouping of objects in the reaching task could be solely due to the fact that all the fruits were round but not due to the fact that they were semantically similar. This way, we controlled for object shape as a main cue in the reaching task and chose triads that tended to be close in similarity (natural: *M*_distance_ = 0.29, *SEM* = 0.04; man-made: *M*_distance_ = 0.33, *SEM* = 0.04) as well as object category similarity (natural: *M*_distance_ = 0.25, *SEM* = 0.04; man-made: *M*_distance_ = 0.24, *SEM* = 0.04) but farther away in shape similarity (natural: *M*_distance_ = 0.57, *SEM* = 0.04; man-made: *M*_distance_ = 0.53, *SEM* = 0.05). Figure 1C shows average pairwise distances for 4 groups of triads. The man-made and natural objects selected for the reaching task tended to have smaller pairwise distances than other randomly selected triads for the natural semantic cluster [*similarity*: *M*_diff1000_ (mean difference from 1000 random triads) = −0.41, *t* = −138.31, *p* < 0.001; *object*: *M*_diff1000_ = − 0.38, *t* = −123.33, *p* < 0.001] and man-made semantic cluster [*similarity*: *M*_diff1000_ = − 0.38, *t* = −126.19, *p* < 0.001; *object*: *M*_diff1000_ = − 0.35, *t* = −111.70, *p* < 0.001].

### Reaching Task

The question we sought to answer was whether object-semantics influence the use of allocentric information for reaching. To answer this question, we conducted a memory-guided reaching task in virtual reality (see Methods). In this task, we placed six objects on a table. Three of these objects belonged to one semantic cluster (natural objects) and three to another (man-made objects). Participants were allowed to freely encode the scene. After a brief mask and a delay, we presented the scene again with one object missing (target object). In all trials except for the no-shift condition (baseline), two objects were shifted horizontally. These two objects either belonged to the same (congruent) or to a different (incongruent) semantic cluster as the target object. Subsequently, on an empty table, the task was to reach to the location of the target object from memory. In the baseline condition, none of the objects were shifted.

To demonstrate the influence of allocentric information, we report allocentric weights as the reaching endpoint errors relative to the maximal expected reaching errors (MERE). Allocentric encoding depending on objects for action would predict that a participant’s reaching endpoint would be significantly shifted in the direction of the object shift. On the other hand, no systematic effect of object shifts would imply that the object had been encoded relative to oneself or other elements in the scene (e.g., edge of the table, start position cube) as opposed to relative to the objects for action.

We used this paradigm with a composition of objects for which we controlled that three of them strongly belonged to the cluster of natural objects and three others to the cluster of man-made objects (Fig. 1C). With similar semantic distances we would expect similar allocentric weights for both semantic clusters.

**Figure 2:**
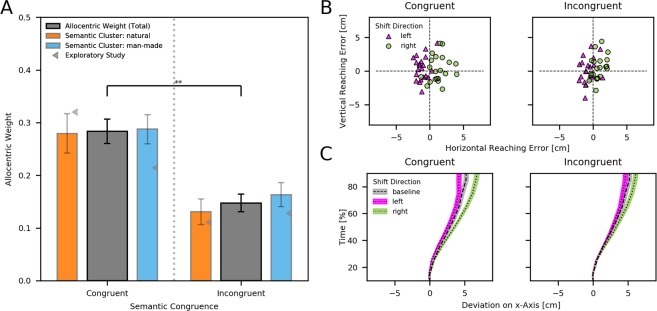
Results of the reaching task. (**A**) Mean allocentric weights grouped by the semantic cluster (natural or man-made) of the target object. Grey triangles indicate group means obtained in the exploratory study with a strong and weak semantic cluster (see Supplementary Information). (**B**) Average horizontal and vertical reaching errors of each participant for both shift directions. (**C**) Average trajectories collapsed across participants for both shift directions and baseline. The trajectories are scaled to the same starting point and represent the middle 90% time-window between movement onset and end of reach. Error bars in A and shaded areas around trajectories in C represent the standard error of mean. **p < 0.01.

The results are depicted in Fig. 2. We found a systematic effect of object shifts with allocentric weights significantly higher than baseline (all tests against zero: *p* < 0.001). More crucially, we found a main effect of semantic congruence [*F*(1, 20) = 11.447, *p* = 0.003, *η*_*p*_^2^ = 0.36], showing higher allocentric weights in the congruent condition (natural: *M* = 0.28, *SEM* = 0.04; man-made: *M* = 0.29, *SEM* = 0.03) as opposed to the incongruent condition (natural: *M* = 0.13, *SEM* =  = 0.02; man-made: *M* = 0.16, *SEM* = 0.02). We neither observed a main effect of semantic cluster (natural vs. man-made) [*F*(1, 20) = 2.350, *p* = 0.141, *η*_*p*_^2^ = 0.11] nor an interaction between semantic congruence and semantic cluster [*F*(1, 20) < 0.001, *p* = 0.978, *η*_*p*_^2^ < 0.01]. The results of the reaching task clearly show that the reaching endpoints systematically deviated in the direction of object shift (Fig. 2B). As illustrated by the descriptive analysis of the movement trajectories (Fig. 2C), these deviations began around halfway through the reach for both semantically congruent and incongruent conditions. The slight shift of the trajectories to the right for both conditions suggests a general rightward bias. This could be caused by a hand effect since participants (who were all right-handed) reached with their right hand, or by the start position of the hand that was aligned to the participants’ body midline. Placing the right index finger on the start position leaves the right hand on the right hand side of the table. Importantly, since both factors were kept constant throughout the experiment, they are unlikely to have influenced our results.

Our results demonstrate a strong effect of semantic congruence on the allocentric weights and no difference between the different semantic clusters. This finding is further strengthened by the results of our exploratory study (see Supplementary Information) where the differences in semantic distance (weak vs. strong) were reflected in significant differences in the allocentric weights (gray triangles in Fig. 2A). Together, the results from these experiments provide convergent evidence that the semantic proximity of our object clusters strongly influences the use of allocentric information for reaching.

## Discussion

We found that humans take semantic information into account when encoding spatial relations of objects for action. This was reflected in reaching endpoints that systematically deviated with shifts of objects in the scene. This effect clearly depended on the semantic congruence between target object and shifted objects. When the target object belonged to the same semantic cluster as the shifted object (semantically congruent), participants integrated allocentric information twice as much as they did when the target and the shifted objects were semantically incongruent. In all conditions, across all semantic clusters, the allocentric weights were significantly above zero, speaking for a general replication of the use of allocentric information for reaching^12,14^. The relatively low allocentric weights are consistent with previous studies which also shifted only a small number of objects e.g.^12,13^. This suggest that participants only partly encoded target objects relative to other nearby objects on the table and made additional use of other allocentric cues or egocentric FoR. This is in line with classic findings showing that egocentric and allocentric FoR are combined for localizing reach targets in space^9–11^.

Our results advance previous work in that we quantify semantic distances and demonstrate how object-semantics influence the extent to which we use allocentric information for goal-directed reaching. By choosing two semantic clusters that are comparable in semantic strength, we show that the probability that an object is encoded relative to other objects increases with its semantic distance to those objects. These findings were further corroborated by the results of our exploratory study (see Supplementary Information for details) suggesting that humans encode objects based on semantic congruence if the brain deems the semantic cluster to be reliable, i.e., less noisy. Previous studies showed that this was the case for other contextual effects such as task relevance^13^.

Our findings could be explained by a Bayesian framework in which egocentric and allocentric information is integrated depending on their relative weighting based on cue reliability and landmark stability^11,32^. The question arises: What makes allocentric information reliable? The presence of multiple landmarks helps us to encode a point in space more accurately. Spatial clusters in a scene can therefore be thought of as reliable information. This was not only demonstrated for memory-guided reaching^12^ but also for wayfinding behavior of animals e.g.^33^. In the context of this study we believe that a strong semantic cluster enhances the creation of a spatial cluster. In other words, we suggest that object-semantics facilitate generating spatial configurations.

The benefit of an object being encoded relative to semantic information in its surroundings was also demonstrated in visual search. For instance, Draschkow, Wolfe and Võ (2014)^34^ compared incidentally with intentionally formed memory representations in naturalistic scenes. Recall memory of scene objects was superior for incidentally encoded objects. Importantly, this benefit was diminished when objects were presented in semantically meaningless scenes. The authors conclude that scene semantics could play a pivotal role in supporting the memorization of scenes during search. On the basis of these findings, further experiments could manipulate the scene background in our paradigm and investigate the effect of semantic distances between scene background and table objects. It is tempting to assume that high-level features of the background determine the semantic processing of objects in a scene. However, recent findings suggest that contextual influences could even arise from low-level features, i.e., summary statistics^35^.

When we selected objects for our reaching task, we tried our best to control for differences in other object properties. For example, we used the multi-arrangement method and asked participants to arrange objects based on their shape. On the one hand, we cannot rule out the influence of other properties, e.g., color or size. Using a variety of objects is what makes our task more natural as opposed to using abstract objects (e.g., cubes or pyramids), which were controlled for such properties and are entirely homogenous. On the other hand, one could imagine that a quite homogenous semantic cluster could be highly reliable (think of three pyramids placed in the environment). In such a scenario, we could expect that objects that are not tightly linked to a cluster are encoded relative to these as well. Future experiments could investigate such “*benefit of incongruence”*.

In the current study we were interested in semantic clustering. However, scene processing is not only affected by semantic but also by syntactical congruence in a scene^36^. We addressed this point by presenting 3D objects in their realistic size and appearance. It remains an open question how syntactical errors (e.g., an apple hanging on a wall instead of a painting) and semantic congruence between objects and their background would interact and affect allocentric coding.

Tools such as virtual reality give us the unique opportunity to investigate these questions while interacting in three-dimensional space which is fully manipulable. Several studies now demonstrate similar effects between virtual reality and classic lab experiments in the broad field of spatial coding^14,37,38^. Through our investigations we gained insight about how we spatially represent objects for action and shed light on the interplay between spatial FoR and human-object interaction. For example, spatial impairments (especially with respect to FoR) can not only be found as a result of age-related cognitive decline, they also serve as cognitive markers that are linked to early stages of Alzheimer’s Disease^39^. To counteract such deficits, it is even more important to understand the basic mechanisms that are involved.

## Methods

### Multi-arrangement task

#### Participants

Fifteen participants with a mean age of 21.33 years (ranging from 18 to 29 years) and normal or corrected-to-normal visual acuity were recruited. All provided informed written consent under an experimental protocol that was approved by ethics board at Justus Liebig University Giessen and in accordance with the Code of Ethics of the World Medical Association Declaration of Helsinki^40^. Participants were compensated with course credits or money.

#### Stimuli

We chose 49 stimuli to come from roughly seven categories: fruits, rocks, office supplies, tools, vegetables, handles, breakfast (see Fig. 1B).

#### Procedure

The experimental procedures were run in MATLAB® Release 2017b (The MathWorks, Inc., Natick, MA) using the multi-arrangement code provided by Kriegeskorte and Mur (2012)^30^ and adapted for the Psychophysics Toolbox^41,42^. The experiments were run on a 17-in. monitor powered by a MacBook Pro (2.8 GHz Intel Core i5) with a resolution of 1280 by 960 pixels. Participants were seated approximately 57 cm from the screen, at which distance 1 cm subtended approximately 1° of visual angle.

We explored similarity between objects by using a multi-arrangement method that, within a testing trial, allows participants to arrange the 2-D distances between a subset of stimuli based on perceived dissimilarity. In the first trial, the multi-arrangement method presents all objects as icons in a circular arrangement around an arena. The icons were placed at regular angular intervals in random order. Participants used the drag and drop controls of the computer’s mouse to organize the icons on the screen based on their similarity. They were told that the distance between two objects represents their similarity, were similar objects are put closer and dissimilar objects are put farther apart. So that participants could more precisely compare the objects, on the right of the arena, the object for the current and last icon selection were increased in their size.

Once the arrangements were complete, the participants pressed the Return key to go to the next trial. The next trials showed a subset of the objects from the first trial based on the lift-the-weakest algorithm described by Kriegeskorte and Mur^30^. The arrangements ended after 12 minutes. On average, 24.2 trials were completed, with the final result being pairwise dissimilarities (in terms of distances) for the set of objects. These dissimilarities were assembled in a representation dissimilarity matrix (RDM), which had a height and width corresponding to the number of objects from Trial 1 and was symmetric along the diagonal.

In the first 12-minute session, we did not explicitly instruct by which similarity criteria to arrange the icons. In the two remaining 12-minute sessions, participants were instructed to arrange the icons based on similarity in object category or shape (in random order). The next trials showed a subset of the stimuli from Trial 1 based on an algorithm that selects similarity with a lower dissimilarity signal-to-noise ratio (i.e., stimuli that tend to be placed closer to one another) and also takes into account the trial cost, i.e., the time taken to arrange the subset^30^. After the arrangements, participants were asked to name the objects to ensure that they were recognized correctly.

### Reaching Task

#### Participants

Twenty-five students of the Justus Liebig University Giessen were initially recruited via university email. The sample size was determined based on an a priori power analysis by means of a Monte Carlo simulation. Since we were not aware of other experiments that similarly investigated the effect in question, we relied on previously recorded data from our lab. The population parameters we chose were therefore obtained from pilot data (*N* = 15) of a similar experiment. With every increment in sample size, we simulated 10,000 full data sets. This resulted in a minimum sample size of 21 participants for a statistical power of 0.80 with respect to the main effect of semantic congruence. Power was defined as the proportion of simulations where the null hypothesis was rejected for a given sample size. All participants provided informed consent and received either course credit or financial compensation. Three were excluded due to an unreliable signal during the calibration of the eye tracker. One was excluded for insufficient stereopsis (graded circle test of the Stereo Fly Test; Stereo Optical Co., Inc., Chicago, IL, USA). This left a remaining sample of 21 participants with a mean age of 23.95 years (range 18–30 years). All were right-handed as assessed via the Edinburgh Handedness Inventory^43^ and reported normal or corrected-to-normal vision. The experimental procedures were approved by the local ethics committee of the Justus Liebig University Giessen and were in accordance with the principles of the Declaration of Helsinki^40^.

#### Apparatus

Participants were seated in front of a table with the similar height as the table in the virtual environment. The table position was aligned to the position of the virtual table. They placed their head on a chin rest which was adjusted to 26 cm above the table edge and aligned to the participant’s body midline. Approximately 30 cm to the left of the chin rest we placed a number pad where one key was used to control the experiment with the left hand. In front of the chin rest (~10 cm from the front edge of the table) we placed a cube of 2 cm^3^ that served as the start position for the right reaching hand. We ran the virtual environment on a Dell Alienware computer with an Intel® Core™ i9 7980XE processor, 32 GB RAM and two NVIDIA® GeForce® GTX™ 1080Ti graphics processing units. The experiment was controlled via Vizard 5.9 (WorldViz, LLC, Santa Barbara, CA, USA) and presented stereoscopically with an HTC Vive HMD at a resolution of 1080 × 1200 pixels per eye and a refresh rate of 90 Hz. Inside the HMD we integrated the Pupil Labs HTC eye-tracker (120 Hz) to control for correct fixation in the critical time windows. Both the fixation control and the stable head position ensured comparability with previous experiments and helped to keep different egocentric representations, such as a gaze- and head-centered reference frames, constant. We measured eye data with a mean angular error of 0.88°. Reach movements were recorded with an Optrotrak Certus (NDI, Waterloo, ON, Canada) at 250 Hz with an infrared marker attached to the right index finger.

#### Stimuli

On every trial, the virtual scene consisted of six objects placed on an 80 × 80 × 86 cm (L × W × H) brown cube that served as a table, three objects for each of the two clusters (Table 1), and a small grey cube that indicated the start position. The table was located 135 cm in front of a beige wall. A red sphere of 1.5 cm radius served as a representation of the position of the right index finger.

**Table 1:** List of objects.

Object	Semantic Cluster	Length [cm]	Width [cm]	Height [cm]
Apple	Natural	8	7	8
Banana	Natural	3	15	6
Pear	Natural	8	8	12
Pencil case	Man-made	7	8	13
Puncher	Man-made	8	12	6
Stapler	Man-made	3	12	8

For each of the six objects we randomly generated four arrangements resulting in 24 arrangements with the following constraints: An object was never placed at the center of the table (fixation cross area) and objects were placed such that they do not overlap. To ensure that target objects were on average equally distant from objects belonging to their own or to the other cluster, we calculated target-cluster distances for every arrangement. On average, the distance between a target and its own cluster (ΔTownC) was 26.68 cm (*SD* = 6.39 cm) while the distance between a target and the other cluster (ΔTotherC) was 24.14 cm (*SD* = 6.53 cm). Post-hoc correlational-analyses confirmed that there was no coding advantage for ΔTownC. This was calculated by subtracting ΔTownC from ΔTotherC and correlating the resulting value with the allocentric weights. To give an example: Assume that a target was an immediate neighbor of its own cluster with ΔTownC = 10 cm and was far away from the other objects with ΔTotherC = 30 cm. Subtracting ΔTownC from ΔTotherC would result in a 20 cm spatial advantage of congruence (because the target is closer to the *own* cluster). If this spatial advantage was a driving factor for the observed results, it should highly correlate with the allocentric weights. None of the conditions were affected by any hypothetical spatial advantage (congruent, *r* = −0.119, *p* = 0.609; incongruent, *r* = 0.027, *p* = 0.907). We used these 24 arrangements five times because of a baseline condition (no shift) and two shift directions (left and right; 5 cm on average) combined with two congruence conditions (whether the target object belonged to the same or to another semantic cluster as the shifted objects). In sum we presented 120 scenes. Additionally, a mask scene was created with 300 grey cubes. These were rendered at an angle of 45° (20 cm side length) and placed randomly covering the participant’s field of view. We used 12 practice trials before each experiment to ensure that participants understood the task. The practice trials did use the same six objects but 12 different spatial arrangements.

#### Procedure

**Figure 3:**
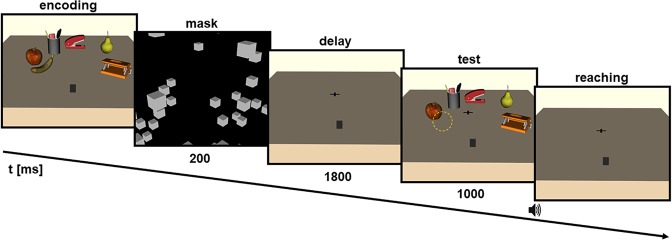
Exemplary trial of the reaching task. Participants freely encoded the scene. After a brief mask and delay, a test scene appeared with one object missing (yellow circle). An auditory go-signal prompted participants to reach to the position of the missing object.

Participants were seated in front of a table. They were told that we would be investigating basic principles of eye-hand-coordination. The time course of a typical trial is depicted in Fig. 3. Each trial consisted of five phases: an encoding, mask, delay, test and reaching phase. In the encoding phase, all six objects were present and participants were allowed to freely explore the environment (self-paced) while resting the right index finger on the start position. Once the participants hit the key with their left hand, the mask appeared (200 ms) followed by a delay that consisted of an empty table (1800 ms) and then the test scene (1000 ms). The test scene was similar to the encoding scene but with the respective target object missing. Here, depending on the condition, two objects in the scene were either shifted or not. Thereafter, the reaching phase was indicated by the onset of a beep tone. Participants were explicitly instructed to reach toward the remembered target position of the encoding scene on an empty table. After a touch was registered, participants were able to start the next trial by pressing the button a second time. During all but the encoding phase participants had to fixate a cross in the center of the table while performing the experiment. We made sure that participants understood the instructions and observed them in an initial practice session. They were not told about the object shifts in the test scene and none of the participants had reported to have seen any shifts.

#### Data Reduction and Statistical Analyses

The data reduction and statistical analyses were conducted in Python 3.7. and R 3.4.3. First we inspected the movement data (2520 trials). We removed all trials with a low number of samples (<20) and trials where the movement trajectory seemed to be out of bounds (e.g., outside or underneath the table) suggesting that the marker camera did not track the marker accurately (~6% affected). Then we filtered all trials where participants started before the go signal (~3% affected). To this end we calculated the movement onset as the first frame in which velocity exceeded 10% of peak velocity. We further filtered all trials where the reaching endpoint deviated more than 2 *SD* in horizontal or vertical direction from the group means (~9% affected). On average, the standard deviations were about 4.73 cm (range 1.63–10.17 cm) in horizontal and 6.51 cm (range 2.23–11.95 cm) in vertical direction. Finally, we inspected gaze data that was preprocessed with a 15-sample median filter. For the fixation periods we calculated the horizontal and vertical gaze range and excluded trials where the gaze range exceeded 4 cm of the table surface in either direction during the fixation period (~12% affected). The excluded trials we report here are not mutually exclusive since in some trials multiple exclusion criteria were reached. We proceeded with all the remaining unaffected trials (~76%).

A key outcome variable in this study are the allocentric weights. Here they represent the ratio between the horizontal reaching error relative to the maximum expected reaching error (MERE). The MERE was calculated as the average horizontal displacement of the shifted objects. Suppose we shift two objects, one by 4 cm and the other by 5 cm to the right, then the MERE would be 4.5 cm. With an exemplary reaching error of +2 cm relative to baseline (no shift condition), the allocentric weight would be 2 ÷ 4.5 = 0.44. This would suggest a 44% influence of allocentric information on the reaching endpoint. It follows the assumption that in a purely allocentric FoR, participants encode the target relative to the table objects. If we shift those objects by 4.5 cm, we maximally expect (hence MERE) a reaching error of 4.5 cm when participants reinstate the geometrical relations, leading to an allocentric weight of 4.5 ÷ 4.5 = 1 or 100%. In the same manner, the difference between the reaching error when objects were shifted leftwards and rightwards was divided by the corresponding difference in MERE to get one allocentric weight across directions.

Normality assumptions were tested with a Shapiro-Wilk test. We conducted a repeated measures analysis of variance to compare group differences in allocentric weights for the within-factors semantic congruence (congruent v. incongruent) and semantic cluster (natural v. man-made). In case of significant interactions, we calculated estimated marginal means for pairwise comparisons. We used one-sample t-tests to check if the allocentric weights significantly differ from zero (=baseline). We applied Bonferroni-Holm correction to account for inflated familywise error rates.

## Supplementary information

Additional_arrangements_and_exploratory_study

## Data Availability

Result data of both experiments can be found at 10.5281/zenodo.2635576.
