# Supplementary material for: Facilitation of allocentric coding by virtue of object-semantics
Source: Sci Rep. 2019 Apr 18;9:6263. doi: 10.1038/s41598-019-42735-4 (PMC6472393; doi:10.1038/s41598-019-42735-4)
Supplement: Supplementary file 1 — Additional_arrangements_and_exploratory_study [file 41598_2019_42735_MOESM1_ESM.pdf]

Supplementary Information

(Additional arrangements and exploratory study)

Facilitation of allocentric coding by virtue of object-semantics

Harun Karimpur*, Yaniv Morgenstern & Katja Fiehler

*Correspondence to harun.karimpur@psychol.uni-giessen.de

1. Supporting figures for the multi-arrangement task

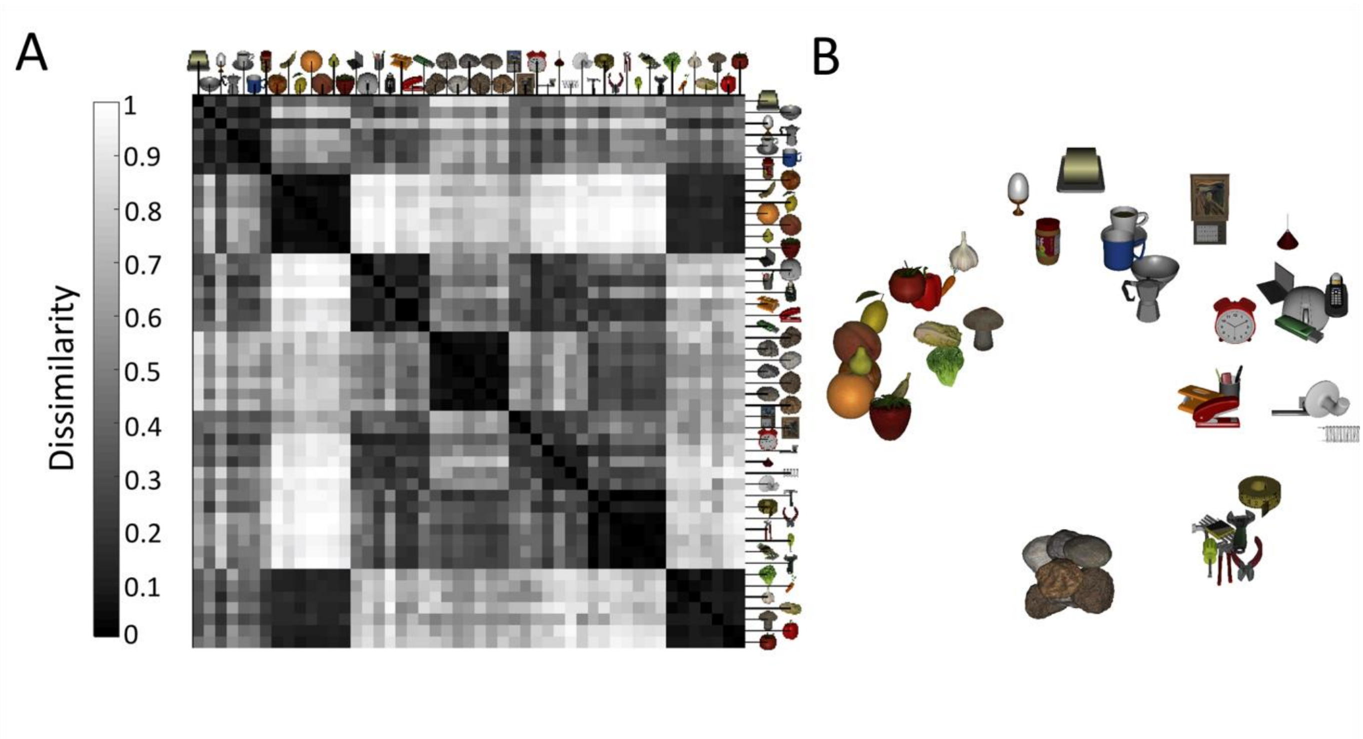

Figure S1. Object arrangements and representation dissimilarity matrix (RDM) for the pooled data in the condition where participants were instructed to arrange stimuli[1)] based on object similarity. For each pair of objects, the RDM (**A**) codes dissimilarity. The objects have been arranged in (**B**) such that their pairwise distances approximately reflect the distances in the RDM (multidimensional scaling; dissimilarity: distances, criterion: metric stress).

[1)] Stove top espresso maker in the supplementary information printed with permission by Jono Moles under a CC BY open access license.

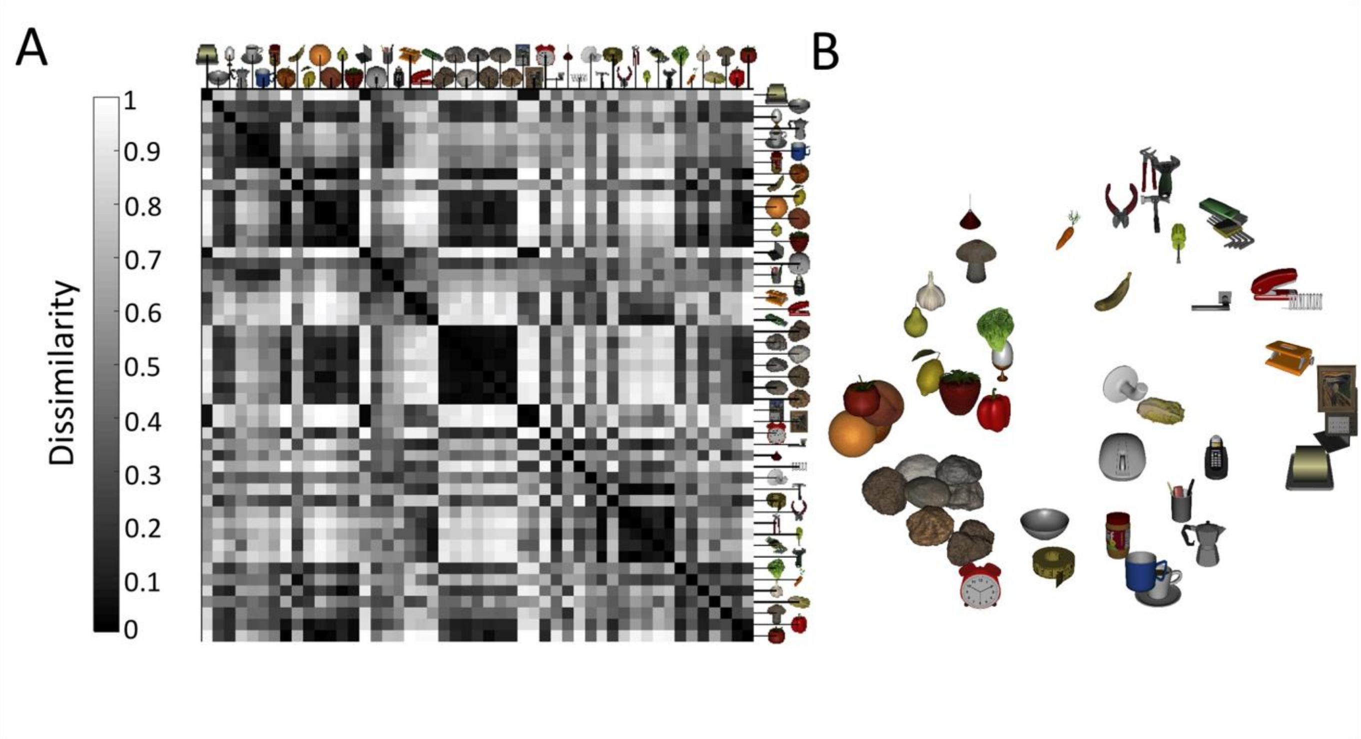

Figure S2. Object arrangements and representation dissimilarity matrix (RDM) for the pooled data in the condition where participants were instructed to arrange stimuli based on shape similarity. For each pair of objects, the RDM (**A**) codes dissimilarity. The objects have been arranged in (**B**) such that their pairwise distances approximately reflect the distances in the RDM (multidimensional scaling; dissimilarity: distances, criterion: metric stress).

2. Exploratory Study

To further test the role of semantics on reaching, we examined data from our exploratory experiment that differed only in the stimuli. The experiment compared a strong semantic cluster (Fig. 1C; natural: apple, orange, pear; similarity $M_{distance}$ = .29, *SEM* = .04) versus a weaker semantic cluster (Fig. 1C; man-made: alarm clock, phone, pencil case; similarity $M_{distance}$ = .58, *SEM* = .06). The results are depicted in Fig. S3. We found again a systematic effect of object shift (all tests against zero: $p < .003$) and a main effect of semantic congruence [$F(1, 15) = 19.993$, $p < .001$, $\eta_p^2 = .57$]. More importantly, we found a significant interaction between semantic congruence and semantic cluster [$F(1, 15) = 5.471$, $p = .034$, $\eta_p^2 = .27$]. Only in case of natural objects (strong semantic cluster) we found that allocentric weights were about twice as high as the allocentric weights in the incongruent condition [$\Delta_{estimate}$ = .158, $SEM_{contrast}$ = .035, $p = .003$]. All other pairwise comparisons yielded no effect (all $p < .2$). In sum, we found that the semantic distance of the object groups interacted with the semantic congruence between target and shifted objects. Only in case of a strong semantic cluster we found an effect of semantic congruence.

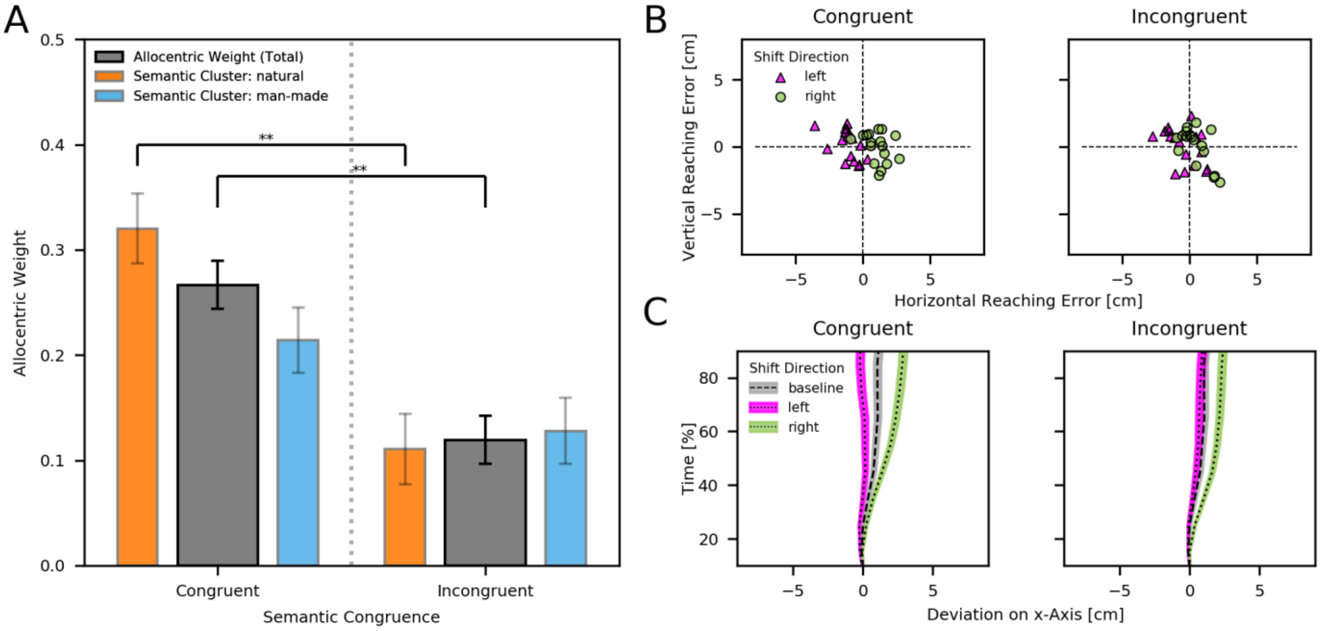

Figure S3. Results of the reaching task comparing a strong semantic cluster (natural) with a weak semantic cluster (man-made). (**A**) Mean allocentric weights grouped by the semantic cluster of the target object. (**B**) Average horizontal and vertical reaching errors of each participant for both shift directions. (**C**) Average trajectories collapsed across participants for both shift directions and baseline. The trajectories are scaled to the same starting point and represent the middle 90% time-window between movement onset and end of reach. Error bars in A and shaded areas around trajectories in C represent the standard error of mean. ** $p < 0.01$.

Eighteen students provided informed consent and received either course credit or financial compensation. One was excluded due to difficulties in calibrating the eye tracker. The experimental protocols were approved by the local ethics committee of the Justus Liebig University Giessen and were in accordance with the principles of the Declaration of Helsinki. The methodological approach was similar to the one described in the Methods section of the reaching task. At the time of the exploratory study we presented the virtual environment on an Oculus Rift DK2 HMD at a resolution of 960 x 1080 pixels per eye and a refresh rate of 75 Hz. Eye movement were recorded

using an infrared camera based eye tracker integrated in the HMD by SMI (SensoMotoric Instruments GmbH, Teltow, Germany). Here we created 8 arrangements and used each arrangement for 3 targets. We combined 2 semantic congruence conditions (congruent, incongruent) with 3 shift conditions (left, right, no shift) resulting in 144 trials.
